# High Sensitivity Shortwave Infrared Photodetector Based on PbS QDs Using P3HT

**DOI:** 10.3390/nano11102683

**Published:** 2021-10-12

**Authors:** Jin Beom Kwon, Maeum Han, Dong Geon Jung, Seong Ho Kong, Daewoong Jung

**Affiliations:** 1 Advanced mechatronics R&D Group, Korea Institute of Industrial Technology (KITECH), Daegu 42994, Korea; jinbum0301@kitech.re.kr (J.B.K.); jdg8609@kitech.re.kr (D.G.J.); 2 School of Electronic and Electrical Engineering, College of IT Engineering, Kyungpook National University, 80 Daehakro, Daegu 41566, Korea; mehan@knu.ac.kr

**Keywords:** infrared, SWIR photodetector, PbS, quantum dots, P3HT

## Abstract

Shortwave infrared (SWIR) photodetectors are being actively researched for their application in autonomous vehicles, biometric sensors, and night vision. However, most of the SWIR photodetectors that have been studied so far are produced by complex semiconductor fabrication processes and have low sensitivity at room temperature because of thermal noise. In addition, the low wavelength band of the SWIR photodetectors currently used has a detrimental effect on the human eye. To overcome these disadvantages, we propose a solution-processed PbS SWIR photodetector that can minimize harmful effects on the human eye. In this study, we synthesized PbS quantum dots (QDs) that have high absorbance peaked at 1410 nm and fabricated SWIR photodetectors with a conductive polymer, poly(3-hexylthiophene) (P3HT), using the synthesized PbS QDs. The characteristics of the synthesized PbS QDs and the current-voltage (I-V) characteristics of the fabricated PbS SWIR photodetectors were measured. It was found that the maximum responsivity of the optimized PbS SWIR photodetector with P3HT was 2.26 times that of the PbS SWIR photodetector without P3HT. Moreover, due to the high hole mobility and an appropriate highest occupied molecular orbital level of P3HT, the former showed a lower operating voltage.

## 1. Introduction

Photodetectors are widely used in various cutting-edge industries, such as the military industries, autonomous vehicles, and biometric sensors. Among them, shortwave infrared (SWIR) photodetectors have attracted considerable attention owing to their high penetration and low scattering. Due to these characteristics, SWIR photodetectors are able to offer better image resolution than visible-light photodetectors at night or in bad weather and many kinds of SWIR photodetectors have been studied. However, most reported InGaAs- and GaAs-type SWIR photodetectors have a high barrier to market entry because of their monopoly, and they necessitate metal organic chemical vapor deposition and two-dimensional epitaxial growth. Therefore, their manufacture is, thus, an expensive and complicated process; moreover, a cooling device is essential because of the high thermal noise at room temperature [1,2,3,4,5,6,7]. In addition, the currently applied SWIR sensor detects infrared rays in a low wavelength band under 1400 nm, which has a detrimental effect on the human eye. To overcome these problems, many researchers are studying solution-processed SWIR photodetectors that do not require complex semiconductor fabrication processes and an additional cooling system [6,8]. In particular, studies based on QDs are actively being conducted. Most QDs, which are semiconductor compounds, can be fabricated by the colloidal method and adapted to many devices through solution processes, such as ink-jet printing and spin coating [9,10,11,12]. The method and process are expected to be low-cost and simple and can be applied to fabricate flexible devices [13,14,15]. Moreover, due to the quantum confinement effect, because the wavelength band can be easily adjusted by controlling the core size of the QDs, it is possible to synthesize QDs that are less harmful to the human eye [16]. Among the many types of QDs, PbS QD-based SWIR photodetectors that cover the entire SWIR wavelength range have been reported, but they have difficulty detecting weak signals because of their low sensitivity. Poly(3-hexylthiophene) (P3HT) which is a conductive polymer has been reported to have high hole mobility and an appropriate highest occupied molecular orbital (HOMO) level (−5.0 eV) and can improve the quality of a thin film. Therefore, it is widely applied to solution process devices, such as solar cells and energy harvesting, and it is possible to improve the properties by applying it to a photodetector [17,18,19,20,21]. In this paper, we propose a highly sensitive PbS QD-based SWIR photodetector fabricated using a spin-coating process that can operate at room temperature. To reduce the harm to the human eye, QDs capable of absorbing infrared rays in the 1410 nm wavelength band were synthesized. To improve the sensitivity of the SWIR photodetector, we adapted P3HT as a hole extraction layer (HEL) which decreased the band gap difference between the PbS QDs and the indium thin oxide (ITO) electrode and increased the hole mobility. The photodetector was fabricated by a simple solution process, and it operated at room temperature without a cooling device, thereby overcoming the drawbacks of conventional infrared (IR) photodetectors. Compared with a PbS SWIR photodetector without a P3HT layer, an optimized PbS SWIR photodetector with a P3HT layer not only showed lower-power operation but also had a maximum responsivity, which was 2.26 times greater. 

## 2. Experimental

### 2.1. Materials

Sulfur (99.998%, Sigma-Aldrich, St. Louis, MO, USA), oleylamine (70%, Sigma-Aldrich, St. Louis, MO, USA), lead chloride (99.999%, Sigma-Aldrich, St. Louis, MO, USA), N_2_ gas flow (99.999%, Daeyang Gas Inc., Busan, Korea), trioctylphosphine (97%, Sigma-Aldrich, St. Louis, MO, USA), ethanol (99%, Sigma-Aldrich, St. Louis, MO, USA), toluene (99.8%, Sigma-Aldrich, St. Louis, MO, USA), P3HT (regioregular, Sigma-Aldrich, St. Louis, MO, USA) and aluminum (ReagentPlus, beads, 5–15 mm, 99.9% trace metals basis, Sigma-Aldrich, St. Louis, MO, USA) were used as received.

### 2.2. Synthesis of Colloidal PbS QDs

By the quantum confinement effect which is a physical phenomenon in which the band gap of QDs changes as a function of the nanoparticle (NP) size, we can control the wavelength band of QDs by manipulating the size of their NPs. In this study, we synthesized PbS QDs with an absorption wavelength band of 1410 nm using a colloidal method [22,23,24,25,26]. “Colloid” refers to a dispersion of particles that are larger than molecules and ions in a gas or liquid with the particles having at least one dimension between approximately 1 nm and 1 µm. First, a mixture of 0.36 mmol of sulfur (S) and 0.24 mL of oleylamine (OLA) was stirred at room temperature for 30 min. Next, a mixture of 1–3 mM mmol of lead chloride (PbCl_2_) and 5 mL of OLA was stirred in a three-neck flask at room temperature under N_2_ gas flow for 30 min, heated to 155 °C for 1 h, and cooled to 120 °C under vacuum for 20 min. Subsequently, the prepared S stock solution and 225 μL of trioctylphosphine (TOP) were quickly injected into the three-neck flask under N_2_ gas flow. After allowing the chemical reaction to proceed at 100 °C for 30 min, the three-neck flask was cooled to room temperature. To remove the excess reagent that had not been incorporated during the synthesis, we centrifuged (FLETA–5, Hanil Scientific Inc., Gimpo, Korea) a mixture of the synthesized PbS QDs and 20 mL of ethanol at 4000 rpm for 10 min. Finally, the purified PbS QDs were dispersed in toluene at a concentration of 30 mg/mL. 

### 2.3. Device Fabrication

**Figure 1:**
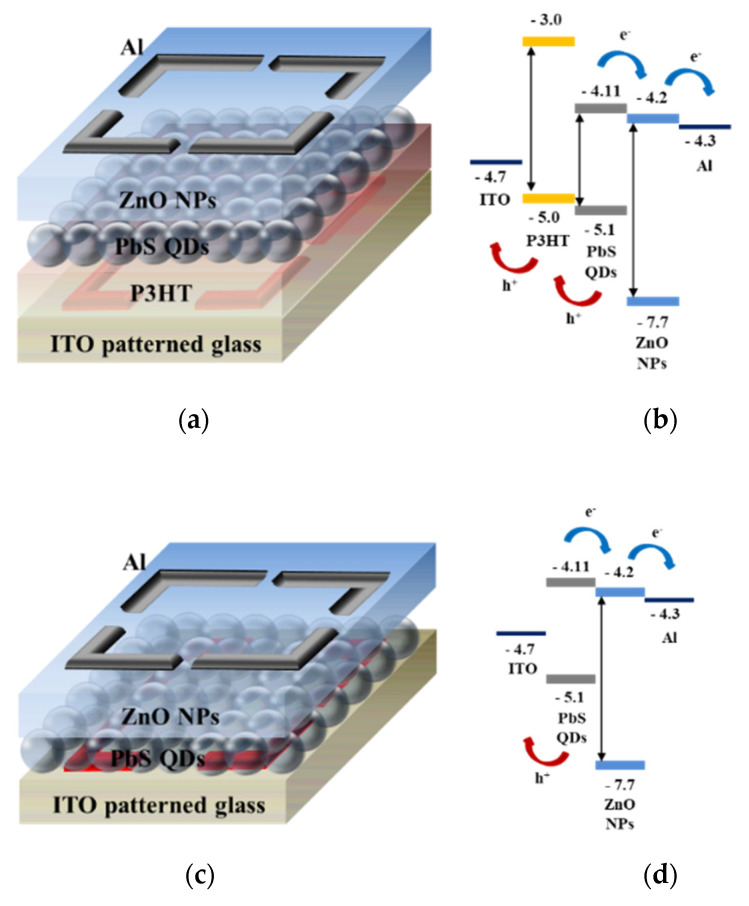
(**a**) Structure and (**b**) energy band diagram of PbS SWIR photodetector without P3HT and (**c**) structure and (**d**) energy band diagram of PbS SWIR photodetector with P3HT.

The PbS SWIR photodetectors were fabricated on glass substrates coated with a patterned indium thin oxide (ITO) anode by spin-coating (LT–MS 200, LTS, Gyeonggi-do, Korea). The thickness of the ITO anode was approximately 400 Å, and the surface resistance was less than 12 Ω. Initially, to prevent the contamination of the ITO–patterned glass, we cleaned the glass with acetone, methanol, deionized water, and UV/ozone. To fabricate PbS SWIR photodetector with P3HT of 20 mg/mL (SWIR PD 2), 30 mg/mL (SWIR PD3), and 40 mg/mL (SWIR PD 4), we formed an HEL by spin casting P3HT solution and heated at 90 °C on a hot plate for 30 min (when fabricating a photodetector without a P3HT (SWIR PD 1), this process was omitted). P3HT solution with a concentration of 10 mg/mL was prepared by dissolving P3HT in chlorobenzene. Due to its high hole mobility and an appropriate HOMO level of −5.0 eV, the HEL could effectively transfer the holes formed in the photoactive layer. To form the photoactive layer, we coated the synthesized PbS QD solution onto the substrate, and heated for 30 min at 110 °C in vacuum oven (OV-11, JEIO Tech., Daejeon, Korea). To form the electron extraction layer, we coated ZnO NPs solution on the active layer and heated the substrate at 90 °C in a vacuum oven for 30 min. ZnO NPs were synthesized by the sol-gel method which was optimized in our laboratory and they could effectively transfer the electrons formed in the photoactive layer to the aluminum cathode because of their high electron mobility and an appropriate lowest unoccupied molecular orbital level of −4.2 eV [27,28,29,30]. Finally, the Al cathode was deposited via thermal evaporation (OLED system, ULTECH, Daegu, Korea) under high-vacuum using a metal shadow mask. The Al electrode thickness exceeded 150 nm, and the detection area defined by the cross section between the Al cathode and the ITO anode was 9 mm^2^. The current-voltage (I-V) characteristics of the PbS SWIR photodetectors were determined using a parameter analyzer (B1500A, Agilent, Santa Clara, CA, USA). Figure 1 shows the structure and energy band diagrams of the fabricated photodetectors. 

## 3. Results

### 3.1. Characteristics of Synthesized PbS QDs

**Figure 2:**
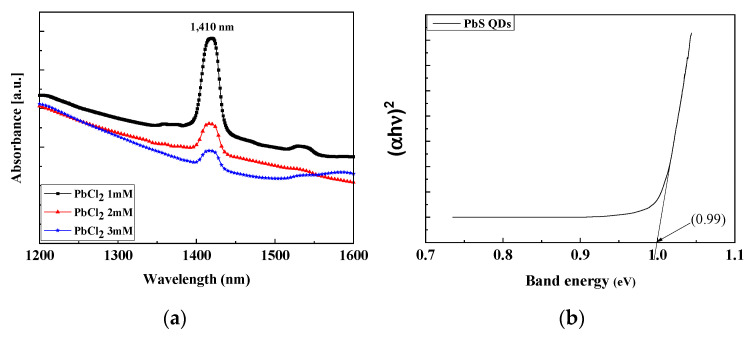

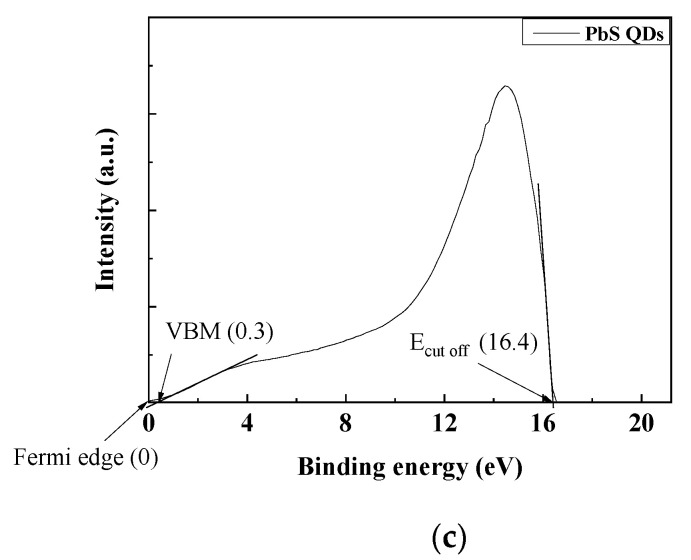
(**a**) Absorption property; (**b**) band gap energy; (**c**) UPS spectrum of the synthesized PbS QDs.

The absorption spectra of the synthesized PbS QDs were measured using a spectrometer (Cary 5000 UV-Vis-NIR spectrophotometer, Agilent, Santa Clara, CA, USA). As shown in Figure 2a it was confirmed that the synthesized PbS QDs have an absorption peak at a wavelength (λ) of 1410 nm and the highest intensity at a PbCl_2_ of 1 mmol. These results indicate that the synthesized PbS QDs could absorb 1410 nm wavelength light and generate electron-hole pairs (EHPs) when irradiated with IR light. As shown in Figure 2b the bandgap energy (*E_g_*) can be determined by extrapolating the linear region down to zero absorbance in the longer wavelength region and was found to be 0.99 eV using Equation (1).

(1) $$E_{g}\left(\mathrm{eV}\right)=\frac{1242}{\lambda \;\left(\mathrm{nm}\right)}$$

The bandgap energy of the materials, measured from the optical absorption plots, can be related to the absorbance (*α*), by the following Equation (2)

(2) $$\alpha h\nu =A{\left(h\nu -E_{g}\right)}^{1/2}$$

where *hν* is the photon energy, and A is the proportionality constant. 

To confirm the fermi level (*E_F_*) and valence band of the synthesized PbS QDs, ultraviolet photoelectron spectroscopy (UPS, NEXSA, ThermoFisher, Waltham, MA, USA) spectrum was measured and they were calculated the following equation.

(3) $$\mathrm{Work}\;\mathrm{Function}=h\nu -(E_{cut\;off}-E_{F})$$

where *hν* is the photon energy, *E_cut off_* is the cut off energy and *E_F_* is the fermi edge.

As shown in Figure 2c, *E_cut off_* was 16.4 eV and *E_F_* was 0 eV and the used photon energy was 21.2 eV. By calculating these through Equation (3), the work function is confirmed to be 4.8 eV. In semiconductors, the difference as much as the work function from the vacuum level is defined as the fermi level, so the fermi level of the synthesized PbS QDs is confirmed to be −4.8 eV. In addition, since the valence band maximum (VBM) representing the energy gap from the Fermi level to the valence band (*E_v_*) is 0.3 eV, it is confirmed that *E_v_* is −5.1 eV. Additionally, since the band gap energy of PbS QDs calculated from the above absorbance characteristics was 0.99 eV, it is confirmed that its conduction band (*E_c_*) was −4.11 eV [31,32,33].

**Figure 3:**
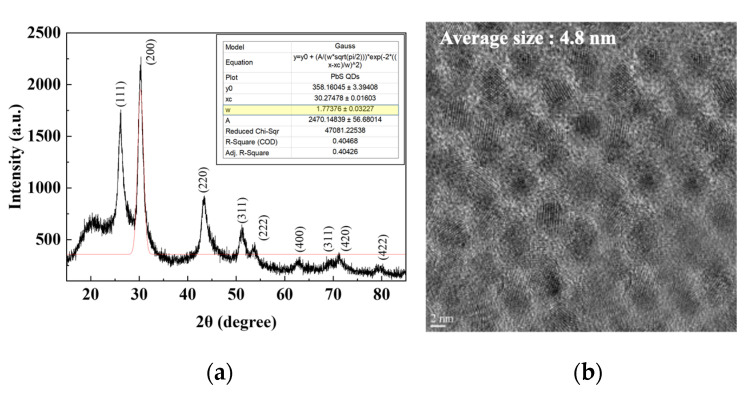
(**a**) XRD analysis and (**b**) TEM image of the synthesized PbS QDs.

X-ray diffraction (XRD; D8-DISCOVER, Bruker AXS, Billerica, MA, USA) analysis was performed to confirm the crystal structure of the PbS QDs. The PbS QD sample for XRD analysis was prepared by spin-coating a PbS QD solution with a concentration of 30 mg/mL on a 10 × 10 mm^2^ glass substrate at a speed of 1000 rpm for 30 s, followed by annealing at 110 °C for 30 min. The XRD results are shown in Figure 3a It was confirmed that the synthesized PbS QDs had the same lattice peak as the reported PbS QDs. To calculate the crystallite size of the PbS QDs from the XRD results, the Scherrer equation was used.

(4) $$D_{hkl}\;\left(\mathrm{nm}\right)=\frac{K\times \lambda }{\beta \cos\theta }$$

where K is the Scherrer constant, *λ* is the wavelength of the X-ray, *β* is the full width at half maximum in radians, and *θ* is the half value between the incident angle and the scattered X-ray wavelength vector [34,35,36]. The PbS QD size calculated from Equation (4) was 4.86 nm. Gaussian fitting was performed to confirm the theta value and FWHM of the peak, and a peak that was accurately fitted was selected. K is 0.94 for spherical crystallites with cubic symmetry, *θ* = 15.1, FWHM = 1.77, *λ* = 0.15418 nm, the wavelength of Cu K-alpha X-rays. Additionally, transmission electron microscopy (TEM; Titan G2 ChemiSTEM Cs Probe, FEI Company, Hillsboro, OR, USA) images were used to observe the synthesized PbS QDs. Samples for TEM analysis were fabricated using a focused ion beam (FIB, NX5000, Hitachi, Krefeld, Germany), which can make thin samples of 100 nm or less using Ga ions. TEM measurements were carried out at a voltage of 200 kV in the BF of the TEM mode using a high-visibility low-background double-tilt holder. As shown in Figure 3b, it was confirmed that the average particle size was 4.8 nm by using ImageJ (National institute of Health, Betheda, MD, USA), which was similar to the particle size calculated by XRD analysis.

### 3.2. Performance of SWIR Photodetectors

**Figure 4:**
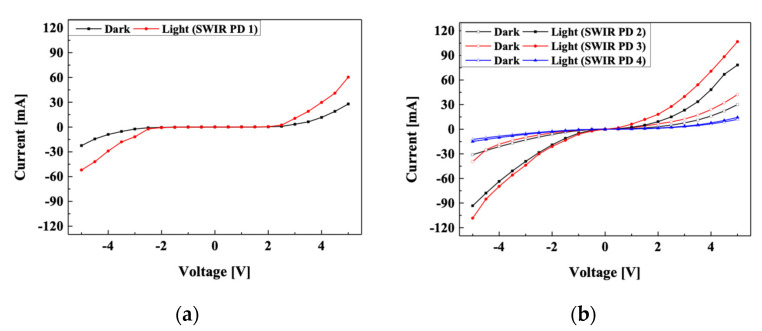
I-V characteristics of (**a**) PbS SWIR photodetector without P3HT (SWIR PD 1) and (**b**) PbS SWIR photodetector with P3HT (SWIR PD 2, 3, 4).

To compare the performance of the fabricated PbS SWIR photodetectors, we measured their I-V characteristics and responsivity. When the PbS SWIR photodetectors were irradiated with IR light, the EHPs generated in the photoactive layer were extracted to the electrodes by an external electric field. The dark current was measured when the IR light source (SL-5, StellarNet Inc., Tampa, FL, USA) was turned off, and the light current was measured when the light source was turned on. The voltage sweep range was from −5 V to 5 V and the output power of the IR light source was 1 W/m^2^. Figure 4a,b show the I-V characteristics of the fabricated PbS SWIR photodetectors. To calculate the fabricated SWIR photodetector from the I-V characteristics, the following equation was used.

(5) $$\mathrm{Responsivity}=\frac{I}{P}$$

where *I* is the current difference between the dark current and light current per unit area, and *P* is the output power of the light source per unit area. In the case of the PbS SWIR photodetector without a P3HT layer (SWIR PD 1), the largest current difference between the dark current and light current and responsivity was observed at −5 V. The maximum current difference was 23.72 mA, and the corresponding responsivity was calculated to be 2635.6 A/W, obtained by dividing this by the input voltage. In the case of the PbS SWIR photodetector with a P3HT layer, SWIR PD 3 in which 20 mg/mL of P3HT was applied showed the best characteristics. The maximum current difference was 53.02 mA at −5 V, and the calculated responsivity was confirmed to be 5891.1 A/W, which is 2.26 times higher than that of SWIR PD 1. This is because when 20 mg/ml of P3HT was applied, the holes generated in the active layer were extracted well to anode. On the other hand, SWIR PD 4, to which 30 mg/mL of P3HT was applied showed a rather low current characteristic. This is because the intensity of the IR light reaching the active layer was greatly reduced according to the concentration of the P3HT layer. In the case of SWIR PD 3, although the intensity of light was decreased by P3HT, it was confirmed that a large current change was caused by the improvement of hole mobility by P3HT. In the case of PD 4, as the concentration of P3HT was too high and the intensity of light irradiated to the PbS QD layer was significantly reduced, EHP generation is greatly reduced and current change was reduced.

Therefore, the device with a P3HT layer could be driven with a lower voltage, which is advantageous for low-power operation because of the high hole mobility and reduction in the band gap between ITO and PbS QDs by an appropriate HOMO level of P3HT. 

## 4. Conclusions

We synthesized PbS QDs by a colloidal method and fabricated a solution-processed PbS SWIR photodetector by adapting a P3HT layer to improve its sensitivity. To confirm the synthesized PbS QDs, absorption spectra, XRD and UPS analyses were performed and TEM was measured. The synthesized PbS QDs have an absorption band maximum at 1410 nm wavelength, and, thus, could detect infrared rays that are less harmful to the human eye when used in sensor systems. In addition, the synthesized PbS QDs have a LUMO level of −4.11 eV and HOMO level of −5.1 eV with a bandgap energy of 0.99 eV. At the HOMO level, the generated holes are extracted toward the anode through the HOMO level of HEL, and at the LUMO level, electrons are extracted toward the cathode through an LUMO level of EEL, which causes improved electrical conductivity. The I-V characteristics were measured to compare the fabricated PbS SWIR photodetectors. The light current was measured with IR light turned on and the dark current was measured with IR light turned off while sweeping the voltage from −5 to 5 V. The maximum responsivity of the device with P3HT was 5891.1 A/W and that of the device without P3HT was 2635.6 A/W. As a result, the maximum responsivity of the PbS SWIR photodetector with a P3HT layer was 2.26 times greater. Moreover, unlike the PbS SWIR photodetector without a P3HT layer, the PbS SWIR photodetector with a P3HT layer could be operated at a low voltage (±0.5 V) because its dead zone was significantly reduced. This is because P3HT reduces the large band gap between the PbS QDs and the ITO, so that the hole generated in the active layer are smoothly discharged by the improved hole mobility.

## Data Availability

The data presented in this study are available on request from the corresponding author.

## References

[1] García de Arquer F.P., Armin A., Meredith P., Sargent E.H. (2017). Solution-processed semiconductors for next-generation photodetectors. Nat. Rev. Mater..

[2] Konstantatos G., Howard I., Fischer A., Hoogland S., Clifford J., Klem E., Levina L., Sargent E.H. (2006). Ultrasensitive solution-cast quantum dot photodetectors. Nature.

[3] Michel E., Xu J., Kim J.D., Ferguson I., Razeghi M. (1996). InSb infrared photodetectors on Si substrates grown by molecular beam epitaxy. IEEE Photonics Technol. Lett..

[4] Graf M., Scalari G., Hofstetter D., Faist J. (2004). Terahertz range quantum well infrared photodetector. Appl. Phys. Lett..

[5] Wu W., Bonakdar A., Mohseni H. (2010). Plasmonic enhanced quantum well infrared photodetector with high detectivity. Appl. Phys. Lett..

[6] Liu H.C., Li J., Thompson J.R., Wasilewski Z.R., Buchanan M., Simmons J.G. (1993). Multicolor voltage-tunable quantum-well infrared photodetector. IEEE Electron Device Lett..

[7] Gunapala S.D., Levine B.F., Ritter D., Hamm R., Panish M.B. (1991). InGaAs/InP long wavelength quantum well infrared photodetectors. Appl. Phys. Lett..

[8] Kim E.T., Madhukar A., Ye Z., Campbell J.C. (2004). High detectivity InAs quantum dot infrared photodetectors. Appl. Phys. Lett..

[9] Qian L., Zheng Y., Xue J., Holloway P.H. (2011). Stable and efficient quantum-dot light-emitting diodes based on solution-processed multilayer structures. Nat. Photonics.

[10] Chuang C.H.M., Brown P.R., Bulović V., Bawendi M.G. (2014). Improved performance and stability in quantum dot solar cells through band alignment engineering. Nat. Mater..

[11] Hansen J.A., Wang J., Kawde A.N., Xiang Y., Gothelf K.V., Collins G. (2006). Quantum-dot/aptamer-based ultrasensitive multi-analyte electrochemical biosensor. J. Am. Chem. Soc..

[12] Li M., Zhou D., Zhao J., Zheng Z., He J., Hu L., Xia Z., Tang J., Liu H. (2015). Resistive gas sensors based on colloidal quantum dot (CQD) solids for hydrogen sulfide detection. Sens. Actuators B Chem..

[13] Liang R., Yan D., Tian R., Yu X., Shi W., Li C., Wei M., Evans G.D., Duan X. (2014). Quantum dots-based flexible films and their application as the phosphor in white light-emitting diodes. Chem. Mater..

[14] Tanabe K., Watanabe K., Arakawa Y. (2012). Flexible thin-film InAs/GaAs quantum dot solar cells. Appl. Phys. Lett..

[15] Liu H., Li M., Voznyy O., Hu L., Fu Q., Zhou D., Xia Z., Sargent E.H., Tang J. (2014). Physically flexible, rapid-response gas sensor based on colloidal quantum dot solids. Adv. Mater..

[16] Kim J.S., Kang B.H., Jeong H.M., Kim S.W., Xu B., Kang S.W. (2018). Quantum dot light emitting diodes using size-controlled ZnO NPs. Curr. Appl. Phys..

[17] Sonar P., Sreenivasan K.P., Maddanimath T., Vijayamohanan K. (2006). Erratum: Comparative behavior of CdS and CdSe quantum dots in poly(3-hexylthiophene) based nanocomposites. Mater. Res. Bull..

[18] Gemayel M.E., Narita A., Dossel L.F., Sundaram R.S., Kiersnowski A., Pisula W., Hansen M.R., Ferrari A.C., Orgiu E., Feng X. (2006). Graphene nanoribbon blends with P3HT for organic electronics. Nanoscale.

[19] Seidler N., Lazzerini G.M., Destri G.L., Marletta G. (2013). Enhanced crystallinity and film retention of P3HT thinfilms for efficient organic solar cells by use of preformed nanofibers in solution. J. Mater. Chem..

[20] Jin S.H., Naidu B.V.K., Jeon H.S., Park S.M., Kim S.C., Lee J., Gal Y.S. (2017). Optimization of process parameters for high-efficiency polymer photovoltaic devices based on P3HT:PCBM system. Sol. Energy Mater. Sol. Cells.

[21] Moreels I., Lambert K., Smeets D., Muynck D., Nollet T., Martins J., Vanhaecke F., Vantomme A., Delerue C., Allan G. (2009). Size-Dependent Optical Properties of Colloidal PbS Quantum Dots. ACS Nano.

[22] Zhao H., Chaker M., Wu N., Ma D. (2011). Towards controlled synthesis and better understanding of highly luminescent PbS/CdS core/shell quantum dots. J. Mater. Chem..

[23] Sargent E.H. (2004). Size-tunable infrared (1000–1600 nm) electroluminescence from solution-processible PbS quantum dot nanocrystals: Towards monolithic optoelectronic integration on silicon. J. Mod. Opt..

[24] Yang X., Ren F., Wang Y., Ding T., Sun H., Ma D., Sun X.W. (2017). Iodide capped PbS/CdS core-shell quantum dots for efficient long-wavelength near-infrared light-emitting diodes. Sci. Rep..

[25] Nam M.W., Lee T.Y., Kim S.W., Kim S.W., Kim S.W., Lee K.K. (2014). Two strategies to enhance efficiency of PbS quantum dot solar cells: Removing surface organic ligands and configuring a bilayer heterojunction with a new conjugated polymer. J. Mater. Chem..

[26] Nam M.W., Park J.P., Kim S.W., Lee K.K. (2014). Broadband-absorbing hybrid solar cells with efficiency greater than 3% based on a bulk heterojunction of PbS quantum dots and a low-bandgap polymer. J. Mater. Chem. A.

[27] Lin K.F., Cheng H.M., Hsu H.C., Lin L.J., Hsieh W.F. (2005). Band gap variation of size-controlled ZnO quantum dots synthesized by sol-gel method. Chem. Phys. Lett..

[28] Kim O.S., Kang B.H., Lee J.S., Lee S.W., Cha S.H., Lee J.W., Kim S.W., Kim S.H., Kang S.W. (2016). Efficient quantum dots light-emitting devices using polyvinyl pyrrolidone-capped ZnO nanoparticles with enhanced charge transport. IEEE Electron Device Lett..

[29] Sun D., Wong M., Sun L., Li Y., Miyatake N., Sue H.J. (2007). Purification and stabilization of colloidal ZnO nanoparticles in methanol. J. Sol-Gel Sci. Technol..

[30] Kwon J.B., Kim S.W., Park C.E., Kim O.S., Xu B., Bae J.H., Kang S.W. (2019). Uncooled short-wave infrared sensor based on PbS quantum dots using ZnO NPs. Nanomaterials.

[31] Maulu A., Rodríguez-Cantó P.J., Navarro-Arenas J., Abargues R., Sanchez-Royo J.F., Garcia-Calzada R., Martinez-Pastor J.P. (2016). Strongly-coupled PbS QD solids by doctor blading for IR photodetection. RSC Adv..

[32] Zhang D., Song J., Zhang J., Wang Y., Zhang S., Miao X. (2013). A facile and rapid synthesis of lead sulfide colloidal quantum dots using in situ generated H_2_S as the sulfur source. CrystEngComm.

[33] Mote V., Purushotham Y., Dole B. (2012). Williamson-Hall analysis in estimation of lattice strain in nanometer-sized ZnO particles. J Theor. Appl. Phys..

[34] Gao J., Jeong S.H., Lin F., Erslev P.T., Semonin O.E., Luther J.M., Beard M.C. (2013). Improvement in carrier transport properties by mild thermal annealing of PbS quantum dot solar cells. Appl. Phys. Lett..

[35] Zhong X., Liu S., Zhang Z., Li L., Wei Z., Knoll W. (2004). Synthesis of high-quality CdS, ZnS, and Zn_x_Cd_1−x_S nanocrystals using metal salts and elemental sulfur. J. Mater. Chem..

[36] Pacholski C., Kornowski A., Weller H. (2002). Self-Assembly of ZnO: From Nanodots to Nanorods. Angew. Chem. Int. Ed..

